# Realizing low voltage-driven bright and stable quantum dot light-emitting diodes through energy landscape flattening

**DOI:** 10.1038/s41377-024-01727-4

**Published:** 2025-01-16

**Authors:** Yiting Liu, Yingying Sun, Xiaohan Yan, Bo Li, Lei Wang, Jianshun Li, Jiahui Sun, Yaqi Guo, Weipeng Liu, Binbin Hu, Qingli Lin, Fengjia Fan, Huaibin Shen

**Affiliations:** 1 Key Laboratory for Special Functional Materials of Ministry of Education, National & Local Joint Engineering Research Center for High-efficiency Display and Lighting Technology, Henan University, 475004 Kaifeng, China; 2 Hefei National Laboratory for Physical Sciences at the Microscale and Department of Modern Physics, CAS Key Laboratory of Microscale Magnetic Resonance, Synergetic Innovation Center of Quantum Information and Quantum Physics, University of Science and Technology of China, 230026 Hefei, China

**Keywords:** Quantum dots, Inorganic LEDs

## Abstract

Solution-processed quantum dot light-emitting diodes (QLEDs) hold great potential as competitive candidates for display and lighting applications. However, the serious energy disorder between the quantum dots (QDs) and hole transport layer (HTL) makes it challenging to achieve high-performance devices at lower voltage ranges. Here, we introduce “giant” fully alloy CdZnSe/ZnSeS core/shell QDs (size ~ 19 nm) as the emitting layer to build high-efficient and stable QLEDs. The synthesized CdZnSe-based QDs reveal a decreased ground-state band splitting, shallow valence band maximum, and improved quasi-Fermi level splitting, which effectively flatten the energy landscape between the QD layer and hole transport layer. The higher electron concentration and accelerated hole injection significantly promote the carrier radiative recombination dynamics. Consequently, CdZnSe-based device exhibits a high power conversion efficiency (PCE) of 27.3% and an ultra-low efficiency roll-off, with a high external quantum efficiency (EQE) exceeding 25% over a wide range of low driving voltages (1.8-3.0 V) and low heat generation. The record-high luminance levels of 1,400 and 8,600 cd m^-2^ are achieved at bandgap voltages of 100% and 120%, respectively. Meanwhile, These LEDs show an unprecedented operation lifetime T_95_ (time for the luminance to decrease to 95%) of 72,968 h at 1,000 cd m^-2^. Our work points to a novel path to flatten energy landscape at the QD-related interface for solution-processed photoelectronic devices.

## Abstract (web summary)

Giant full-alloy QDs flatten the energy disorder between the QD layer and hole transport layer, realizing low voltage-driven bright QLEDs with a T_95_@1000 cd m^-2^ lifetime surpassing 72,000 h.

## Graphical abstract

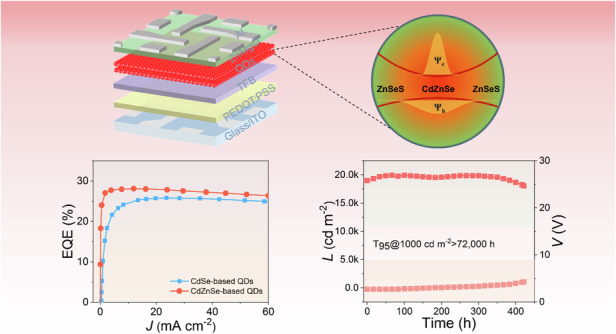

## Introduction

Solution-processed quantum dots (QDs) have attracted enormous interest as an excellent candidate for display and lighting applications owing to their outstanding advantages, such as size-tunable emission wavelengths, near-unity quantum yield (QY), and high color purity^1–6^. QD-based light emitting diodes (QLEDs) have made rapid progress and achieved prominent external quantum efficiencies (EQE) beyond 20% through engineering the chemical synthesis of QDs and device architecture^7–16^. Although QLEDs produce significantly less heat compared to thermal radiation light sources^13^. The suboptimal energy landscape of QLEDs and low thermal conductivity of the functional layer materials can still lead to unavoidable heat accumulation, especially in high-brightness areas, which can result in a roll-off in device efficiency and deterioration in operational lifetime^17–20^.

Although using different types of glass with high thermal conductivity is an effective strategy to enhance heat dissipation, most researchers still aim to reduce heat generation during device operation by manipulating the exciton dynamics process^13,18,21–23^. One of the existing issues is the imbalance of charge injection in the QD-emitting layer. This arises due to the deep valence band position of QDs and the huge difference in carrier mobilities between the electron transport layer (ETL) and hole transport layer (HTL)^11,19,20,24^. Various energy-level alignment engineering strategies, such as doping of ETL, HTL, and nanostructure engineering of QDs, have been attempted to eliminate the exciton quenching and the decomposition of the HTL caused by electron accumulation^9,12,19,24–31^. This also results in the achievement of the turn-on voltage (*V*_on_) below the bandgap voltage. However, at present, the external quantum efficiency (EQE) and luminance of most QLEDs remain relatively low at low voltages. For example, the luminance at bandgap voltage is almost always <1000 cd m^−^², which does not meet the requirements for lighting and display applications^11,13,18^. The valence bands of Cd-based QDs are usually closely packed, in which this degeneracy is incompletely lifted. This leads to the hole population spreading among several states. However, the incomplete degeneracy of the valence band induced by ground-state band splitting leads to energy disorder between QDs and HTL due to the intermediate barrier retarding the thermal relaxation^32^. This results in the high-performance and stable QLEDs obtained from the above strategies requiring a high operating voltage range due to the severe Joule heat generation^13,33–35^.

The exciton fine structure of QDs enables the tailored degeneracy of electronic states at the band edges, spreading the hole population among several states^36–38^. This is not only related to the Auger recombination process and the photoluminescence linewidth but also affects the hole quasi-Fermi level. Recently, we reported a novel large QDs with increased quasi-Fermi-level splitting, effectively achieving enhanced brightness at low driving voltages and reduced device heat generation^13,22^. This inspires us to develop novel large QDs with tunable exciton fine structure splitting to address driving voltage-related device heat generation issues.

Herein, we introduced “giant” full alloy CdZnSe/ZnSeS core/shell QDs (size > 19 nm) with the continuous energy level and delocalized hole wavefunctions. The synthesized CdZnSe-based QDs reveal a suppressed valence-band degeneracy, suppressed ground-state band splitting, and increased electron occupancy concentration, which effectively smooths out the energy disorder at the QD/HTL interface and accelerates the radiative recombination of excitons. Consequently, CdZnSe-based QLEDs exhibit a record-high power conversion efficiency (PCE) of 27.3% and an EQE of surpassing 25% in a wide luminance range of 200–30,000 cd m^−2^ under low driving voltages. High luminance levels of 1400 and 8600 cd m^-2^ are achieved at bandgap voltages of 100% and 120%, respectively. Furthermore, CdZnSe-based devices present a robust operation lifetime (*T*_95_) surpassing 72,000 h at an initial luminance of 1000 cd m^−2^. Our work provides a novel and effective route for the improvement of device stability.

## Results

**Fig. 1:**
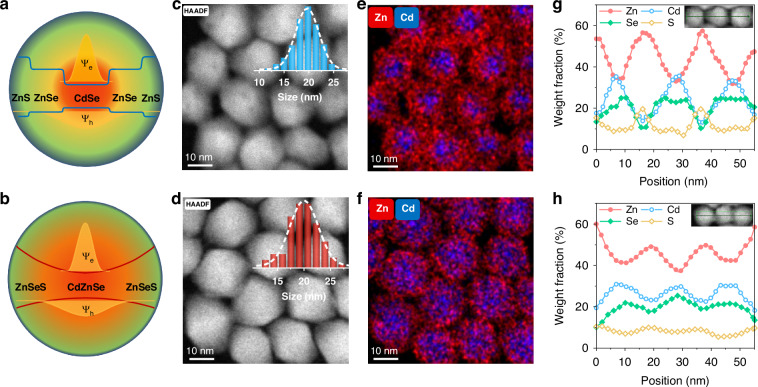
Structural and morphological properties of QDs. Schematic illustrations of QDs with corresponding energy levels and electron/hole wavefunctions for **a** CdSe-based QDs and **b** CdZnSe-based QDs. HAADF scanning transmission electron microscopy images of **c** CdSe-based QDs and **d** CdZnSe-based QDs, insets: the corresponding size distribution histograms. EDS elemental mapping of cadmium and zinc for **e** CdSe-based QDs and **f** CdZnSe-based QDs. EDS elemental line scanning for **g** CdSe-based QDs and **h** CdZnSe-based QDs

We synthesize two types of large-sized QDs with different nanostructures. Traditional CdSe/ZnSe/ZnS core/multishell QDs (named CdSe-based QDs) with graded energy levels present the confined electron and hole wavefunctions in the CdSe core (Fig. 1a). A novel “giant” full alloy CdZnSe/ZnSeS core/shell QDs (named CdZnSe-based QDs) exhibit a fully continuous energy level and delocalized hole wavefunctions, while soothingly releasing the compressive strain (Fig. 1b). Detailed synthesis processes are in the experimental section, and the evolutions of absorption and PL spectra are also monitored (Fig. S1). The synthesized large-seized ZnCdSe/ZnSeS core/shell QDs show a PL emission peak of 621 with 21 nm of FWHM. Transmission electron microscopy (TEM) images of CdSe and CdZnSe-based QDs exhibit that they remain a narrow monodispersity and a similar average size of ~19.6 nm (Fig. S2). To characterize the structure difference between the two QDs, the element distribution is monitored by high-angle annular dark-field (HAADF) scanning transmission electron microscopy element mapping in Fig. 1e, f and S3. The Cd element of CdSe-based QDs mainly distributes in the inner CdSe core, and that of CdZnSe-based QDs is more widely dispersed. Furthermore, the element line scanning spectra of CdZnSe-based QDs reveal that the proportion evolutions of all four elements, especially Cd and Se elements, change more slowly with the radius from the core to the shell, which effectively facilitates hole wavefunctions delocalization. The delocalized exciton wavefunctions usually indicate the weakened exciton-photon coupling and possible ground-state band splitting^36,39^.

**Fig. 2:**
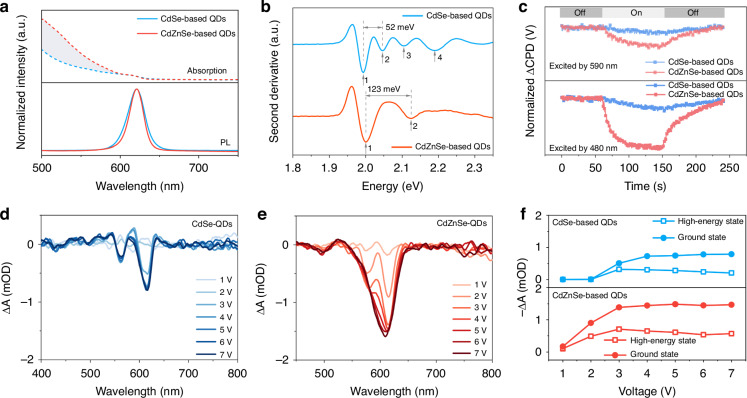
Spectroscopic analysis of two QDs with different valence-band state degeneracy. **a** Absorption and PL spectra. **b** The second derivatives of absorption spectra. **c** ΔCPD evolution using excitation at 590 and 480 nm, the bars at the top represent the on–off status of the excitation light. **d**, **e** EETA spectra at different voltages. **f** The extracted Δ*A* of different bleaching signal peaks for CdSe-based and CdZnSe-based devices

To engineer the degeneracy of valence-band states, Absorption and PL spectra of CdSe and CdZnSe-based QDs are shown in Fig. 2a. All the two QDs present the same PL peak position at 620 nm and almost uniform QY (>95%). CdZnSe-QDs have a higher absorption cross-section below 600 nm, indicating that they maintain a thick CdZnSe alloy layer. Moreover, the PL spectrum of CdZnSe-QDs reveals a narrower full width at half maximum (FWHM) of 22 nm compared to that of CdSe-based QDs (25 nm). Considering the identical size and monodispersity of two QDs, the narrower PL linewidth of CdZnSe-based QDs is associated with the homogeneous broadening of alloying, which is related to the spatial distribution of electron and hole wavefunctions^36,40,41^. Meanwhile, the second derivatives of absorption curves are extracted in Fig. 2b. CdSe-based QDs reveal an obvious heavy hole-light hole splitting, appear the 1P_hh_ and 1S_lh_ states, which is similar to conventional-sized CdSe core–shell QD (Fig. 2b and S4). The energy splitting of ground-state bands is usually caused by compressive strain because of the lattice mismatch in core-shell QDs. CdZnSe-based QDs present the initial ground-state exciton fine structure, namely, 1S_hh_ and 2S_hh_ states, which indicates a suppressed valence-band degeneracy. X-ray diffraction (XRD) patterns of CdSe-based and CdZnSe-based QDs are performed to verify the origin of ground-state band splitting (Fig. S5). Because of the different element distributions, the diffraction peaks of CdZnSe-based QDs shift to a lower angle compared to that of CdSe-based QDs. The crystal plane with the highest diffraction signal intensity for CdZnSe-based QDs is (100), while for CdSe-based QDs, it is the (002) crystal plane. Furthermore, the shift extents of (002) and (110) diffraction peaks are different for the two QDs. The shift difference between (002) and (110) in CdZnSe-based quantum dots (QDs) is smaller than that in CdSe-based QDs. This indicates that CdZnSe-based QDs present a reduced anisotropic contraction due to their smaller shift value, which signifies the smooth compressive strain release.

We attempt to compare the quasi-Fermi-level splitting of two QDs through surface photovoltage spectroscopy using the photo-assisted Kelvin probe technique^22,42,43^. The photo-assisted Kelvin probe technique provides a non-contact method for collecting work function differences by measuring the contact potential difference (CPD) between the probe tip and QD. The change of ΔCPD increases gradually with the increase of excitation light energy in Fig. S6. Compared to CdSe-based QDs, CdZnSe-based QDs exhibit a higher CPD response in the entire excitation wavelength range, which implies a higher electron concentration and a larger quasi-Fermi level splitting (Fig. 2c). With the increase of illumination time by an excitation light at 590 nm, CdZnSe-based QDs exhibit a faster and higher CPD response. Furthermore, although the CPD response of both types of quantum dots increased, the enhancement of CdZnSe-based QDs is more significant under high-energy excitation light at 480 nm. Therefore, CdZnSe-based QDs exhibit a larger quasi-Fermi level splitting under different excitation energies. To further investigate the difference in electron concentration between the two types of QDs, the novel electrically excited transient absorption (EETA) spectroscopy technology is employed for carrier dynamics in Fig. 2d, e^13,26^. There are different changing trends for the absorption differences (Δ*A*) of two electroluminescence devices with CdSe and CdZnSe-based QDs. The bleaching signals of the ground state (~616 nm) and high energy state (~563 nm) are observed. With the increase in voltage, the peak positions of two signal peaks remain almost unchanged for CdSe-based devices. The high energy bleaching signal peak of CdZnSe-based devices gradually moves to the low energy region and merges with the ground state bleaching signal peak. We further extracted the signal intensity values of Δ*A* at two energy states (Fig. 2f). All these bleaching signal values of CdZnSe QLEDs are higher than those of CdSe-based devices under different voltages, which indicates a higher electron population. Meanwhile, the signal values of CdZnSe-based devices rapidly increase and reach saturation in voltage <3 V, which is attributed to the suppressed ground-state band splitting. These results demonstrate that the CdZnSe-based QDs can effectively suppress ground-state band splitting, and increase electron concentration, facilitating the radiative exciton recombination.

**Fig. 3:**
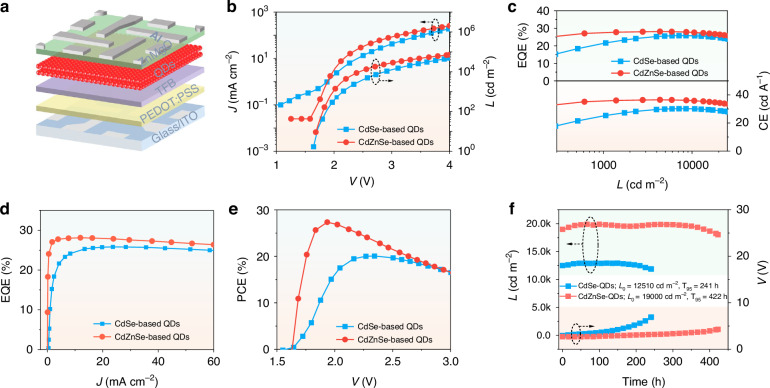
Device structure and performance. **a** Schematic of the device structure. **b** *J–V–L* curves. **c** EQE−*L*−CE curves. **d** EQE−*J* curves. **e** PCE*–V* curves. **f** *L*−Time−*V* curves for two devices using CdSe and CdZnSe QDs

To verify the effect of state degeneracy generated by the ground-state band splitting on device performance, we built QLEDs with the standard structure of ITO/poly-(ethylenedioxythiophene):polystyrenesulfonate (PEDOT:PSS, ~40 nm)/poly{9,9-dioctylfluorene-co-N-[4-(3-methylpropyl)]-diphenylamine} (TFB, ~28 nm)/QDs (~20 nm)/ZnMgO (~60 nm)/Al (Fig. 3a and S7). The energy levels of two QDs are detected by ultraviolet photon spectroscopy (UPS, Fig. S8). The valence band maximum (VBM) value of CdZnSe-based QDs is ~0.08 eV higher than that of CdSe-based QDs, which means a more balanced charge injection. Current density (*J*)−voltage (*V*) curves highlight that CdZnSe-based devices exhibit a significantly reduced leakage current density compared to that of CdSe-based devices (Fig. 3b). As the voltage increases, the EL peak positions of the two types of devices do not show an obvious shift at ~622 nm, exhibiting excellent color stability (Fig. S9). In addition, EL spectra of devices with CdZnSe-based QDs exhibit a narrower FWHM of 22 nm compared to that of devices with CdSe-based QDs. Both types of large QD-based devices exhibit *V*_on_ of ~1.65 V, which is only 0.83 times the bandgap voltage. Consequently, CdSe-based devices show a maximum external quantum efficiency (EQE) of 25.8% and a peak current efficiency (CE) of 30.2 cd A^−1^ at 2.7 V. CdZnSe-based devices exhibit a significantly higher maximum EQE of 28.1% and a maximum CE of 36.5 cd A^−1^ under a lower bias voltage of 2.2 V (Table S1). The EQE of CdZnSe-based devices exceeds 25% in a wide luminance range of 200–30,000 cd m^−2^, indicating a suppressed efficiency roll-off (Fig. 3c). The corresponding current efficiency (CE) also exhibits a puny decline. In contrast, the EQE and CE of CdSe-based devices show an obvious attenuation trend. Further, the suppressed ground-state band splitting and increased electron concentration guarantee that we achieve luminance levels of 1400 and 8600 cd m^−2^ at bandgap voltages of 100% and 120%, respectively. However, the values of CdSe-based QLEDs are only 510 and 3000 cd m^−2^, which are consistent with previous literatures^11–13^. The EQE-*J* curves also clearly show that the CdZnSe-based device exhibits higher EQE at lower current densities due to the effective flattened energy landscape (Fig. 3d). The significantly reduced driving voltage has rightfully enabled red LEDs utilizing CdZnSe-based QDs to achieve a high power conversion efficiency (PCE) of 27.3% (Fig. 3e). The dramatic reduction of the driving voltage range is also beneficial to minimizing heat generation, which is expected to increase the operation lifetime. QLEDs with CdZnSe-based and CdSe-based QDs exhibit *T*_95_ operation lifetimes of 422 h at an initial luminance of 19,000 cd m^−2^ and 241 h at 12,510 cd m^−2^, respectively. The *T*_95_ value at 1000 cd m^−2^ of CdZnSe-based devices is calculated to be about 72,968 h considering an acceleration factor of 1.75 (Fig. S10), which is the highest record of the *T*_95_ lifetime at 1000 cd m^−2^ for red QLEDs (Table S2).

**Fig. 4:**
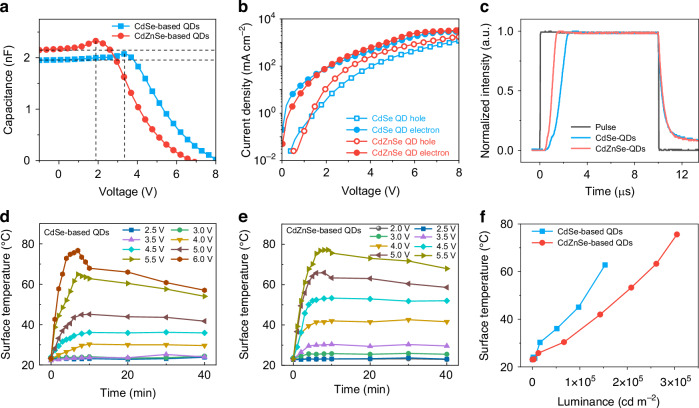
The accelerated radiative recombination of excitons and the evolution of device temperature. **a** C-V curves. **b** *J–V* characteristics of electron-only and hole-only devices. **c** Normalized TrEL with a 3.0 V bias for QLED with CdSe-based and CdZnSe-based QDs. The variation of surface temperature over time under different driving voltages for **d** CdSe-based and **e** CdZnSe-based QDs. **f** Device temperatures under different brightnesses at 10 min

To deeply explore the mechanism of performance improvement, capacitance–voltage (*C*–*V*) curves of devices with CdSe and CdZnSe-based QDs are shown in Fig. 4a. The capacitance value of CdZnSe-based devices first begins to increase at about 0.50 V, which demonstrates that the carrier injection happens. Then, the lower corresponding voltage of the peak capacitance (~1.88 V) is less than that of CdSe-based devices (~3.34 V), which indicates that the carrier dynamics processes, such as injection, transport, accumulation, and recombination, in CdZnSe-based devices, occur at a lower voltage range compared to that of CdSe-based devices. Furthermore, the capacitance value of CdZnSe-based devices is larger than that of CdSe-based devices with a voltage below 2.83 V, which means that it has a higher electron occupancy concentration considering their same sizes. Meanwhile, CdZnSe-based devices also exhibit a lower resistance (Fig. S11). In addition, The *J–V* characteristics of two devices with electron-only and hole-only structures are tested to monitor carrier transport properties (Fig. 4b). CdZnSe-based devices with single-carrier structures reveal a lower *J* value than that of CdSe-based devices under the low voltage range. With the increased voltage, the *J* value of the hole-only device with CdZnSe-based QDs increases rapidly and is close to that of the electron-only device, signifying the enhanced hole injection compared to CdSe-based devices. The transient electroluminescence (TrEL) dynamics technology was employed to monitor the hole injection process (Fig. 4c). The rapidly rising EL intensity of CdZnSe-based devices indicates the rapid injection of holes to satisfy the radiative recombination of carriers.

Furthermore, we studied the effects of reduced driving voltage and increased injection balance on device heat generation. The surface temperature of CdSe-based devices shows almost no variation when the voltage is <3.5 V due to the relatively low electron–hole injection caused by a large barrier. With increasing voltage (<5 V), the surface temperature gradually rises within 10 min and then stabilizes. Further increasing the voltage causes a rapid increase in the surface temperature of the device within a few minutes, due to severe imbalance in charge accumulation. For CdZnSe-based devices, the voltage range for which the surface temperature remains constant is <2.5 V, which is consistent with the low driving voltage. Within the voltage range of 3–4.5 V, the surface temperature of the device shows an increasing trend followed by stabilization over time. The surface temperature of CdZnSe-based devices is below 30 °C within the driving voltage range where EQE exceeds 20%. However, that of CdSe-based devices exceeds 30 °C within the driving voltage range where EQE exceeds 20% (Fig. S12). We further extracted the trends of surface temperature and brightness of the devices during an operation time of 10 min (Fig. 4f), which indicates that the surface temperature of CdZnSe-based devices is significantly lower than that of CdSe-based devices at the same brightness, especially in the high brightness range.

In brief, the ground-state band splitting strategy effectively flattens the energy landscape of the QD/HTL interface, which significantly facilitates the hole injection and accelerates the radiative recombination of excitons, which signifies the enhancement of carrier injection balance. In addition, CdZnSe-based QLEDs reveal a high electron concentration and a reduced and broad driving voltage, which decreases joule heat generation. This significantly inhibits efficiency roll-off and achieves record-high brightness at low drive voltage for CdZnSe-based devices (Fig. S13 and Table S2). These results represent the best values of conventional QLEDs to date, without any light out-coupling structure and engineering of the emitter dipole. We predict that our strategy is also expected to achieve breakthrough progress for blue and green QLEDs, and other photoelectric devices.

## Discussion

In summary, we introduce the novel giant full-alloy CdZnSe/ZnSeS QDs to suppress the valence-band degeneracy, which effectively smooths out the energy disorder at the QD/HTL interface and accelerates the radiative recombination of excitons. The CdZnSe-based QDs reveal a suppressed ground-state band splitting, high electron concentration, and higher VBM value. Consequently, The CdZnSe-based devices exhibit a dramatically reduced driving voltage with EQE larger than 25%, correspondingly, a wide luminance range of 200–30,000 cd m^−2^. In addition to effectively inhibiting the efficiency roll-off, the CdZnSe-based devices show an excellent *T*_95_ operation lifetime exceeding 70,000 h at 1000 cd m^−2^ due to the effectively suppressed joule heat generation. Our strategy provides an efficient path to minimize heat generation and improve device stability.

## Materials and methods

### Materials

Zinc acetate (Zn(ac)_2_, 99.99%), Cadmium oxide (CdO, ≥99.99%), 1-Octadecene (ODE, 90%), Oleic acid (OA, 90%), and Trioctylphosphine (TOP, 90%) were purchased from Sigma-Aldrich. Selenium (99.5%) and Sulfur (99.5%) were obtained from Alfa Aesar. Ethanol (AR, ≥95%), Hexane (AR, ≥97%), and Octane (GC, ≥99%) were obtained from Aladdin. All reagents are used directly as received without further processing.

### Preparation of precursors

Cadmium oleate (Cd(OA)_2_): CdO (20 mmol), OA (20 mL), and ODE (20 mL) were mixed into a 100 mL three-neck flask. The mixture was heated to 240 °C under a nitrogen atmosphere until the solution became clear and transparent, Then, it was cooled down to 60 °C for later use. Zinc oleate (Zn(OA)_2_): Zn(ac)_2_ (20 mmol), OA (20 mL), and ODE (20 mL) were mixed into a 100 mL three-neck flask. The mixture was heated to 150 °C under a nitrogen atmosphere until the solution became clear and transparent, Then, it was cooled down to 100 °C for later use. Se precursor (Se-TOP): Se (15 mmol) was dispersed in TOP (30 mmol) under vigorous ultrasonic conditions. S precursor (S-TOP): S (15 mmol) was dispersed in TOP (30 mmol) under vigorous ultrasonic conditions.

### Synthesis of CdZnSe/ZnSeS QDs

For the synthesis of CdZnSe/ZnSeS core/shell QDs. 2.5 mmol of CdO, 2 mmol of Zn(ac)_2_, 10 mL of OA, and 25 mL of ODE were placed into a 100 mL three-neck flask. Under a nitrogen atmosphere, the temperature was raised to 120 °C and held for 60 min to remove gas, followed by an increase in temperature to 305 °C. The 8 mL of Se-TOP precursor was then rapidly injected, and the reaction was maintained for 90 min. To further grow the alloy CdZnSe core, 4 mL of Cd(OA)_2_ and 8 mL of Zn(OA)_2_ were added to the reaction system. The Se precursor (8 mL) was then added dropwise at a rate of 6 mL h^−1^ at a temperature of 305 °C. To grow the ZnSeS shell, 5 mL of Zn precursor was injected into the reaction system, followed by the simultaneous dropwise addition of 2.5 mL of Se-TOP precursor and 2.5 mL of S-TOP precursor at a rate of 6 mL h^−1^. The reaction system was cooled to room temperature, resulting in the final production of ZnCdSe/ZnSeS QDs. Ethanol and hexane were used for the purification of QDs. The purified QDs can be dispersed in octane for further characterization and device fabrication.

### Synthesis of CdSe/ZnSe/ZnS QDs

CdSe cores with a diameter of 3.5 nm were prepared according to our previously reported recipe. To relieve the lattice stress between the CdSe core and the ZnSe shell, we first grew a CdZnSe shell on the surface of the CdSe core. The temperature of the reaction system was raised to 305 °C. Then over 40 min, 1 mL of Cd(OA)_2_ precursor, 4 mL of Zn(OA)_2_ precursor, and 3 mL of Se-TOP precursor were added dropwise simultaneously. To grow the ZnSe shell, 8 mL of Zn precursor was injected into the reaction system, followed by the simultaneous dropwise addition of 8 mL of Se-TOP precursor at a rate of 6 mL h^−1^. The ZnSeS alloy shell was introduced to further relieve stress. 4 mL of Zn precursor was injected into the reaction system, followed by the simultaneous dropwise addition of 2 mL of Se-TOP precursor and 2 mL of S-TOP precursor at a rate of 6 mL h^−1^. For the outer ZnS shell, 10 mL of Zn precursor was injected into the reaction system, followed by the simultaneous dropwise addition of 10 mL of S-TOP precursor at a rate of 6 mL h^−1^. The reaction system was cooled to room temperature, resulting in the final production of CdSe-based QDs.

## Supplementary information

Supporting Information
