# Supplementary material for: Realizing low voltage-driven bright and stable quantum dot light-emitting diodes through energy landscape flattening
Source: Light Sci Appl. 2025 Jan 16;14:50. doi: 10.1038/s41377-024-01727-4 (PMC11739401; doi:10.1038/s41377-024-01727-4)
Supplement: Supplementary file 1 — Supporting Information [file 41377_2024_1727_MOESM1_ESM.docx]

**Supporting Information**

**Realizing low voltage-driven bright and stable quantum dot light-emitting diodes through energy landscape flattening**

*Yiting Liu,^1#^ Yingying Sun,^1#^ Xiaohan Yan,^2^ Bo Li,^2^ Lei Wang,^1*^ Jianshun Li,^1^ Jiahui Sun,^1^ Yaqi Guo,^1^ Weipeng Liu,^1^ Binbin Hu,^1^ Qingli Lin,^1^ Fengjia Fan,^*2^ Huaibin Shen^1*^*

^1^ Key Laboratory for Special Functional Materials of Ministry of Education, National & Local Joint Engineering Research Center for High-efficiency Display and Lighting Technology, Henan University, 475004 Kaifeng, China.

^2^ Hefei National Laboratory for Physical Sciences at the Microscale and Department of Modern Physics, CAS Key Laboratory of Microscale Magnetic Resonance, Synergetic Innovation Center of Quantum Information and Quantum Physics, University of Science and Technology of China, 230026 Hefei, China.

^#^Yiting Liu and Yingying Sun contributed equally to this work.

*e-mail: wanglei7869@henu.edu.cn; [ffj@ustc.edu.cn](mailto:ffj@ustc.edu.cn); shenhuaibin@henu.edu.cn

**Experimental section**

**Characterization of Materials**

UV-vis absorption spectra and PL spectra were obtained by an Ocean Optics spectrophotometer (model PC2000-ISA). TEM images were photoed by JEOL JEM-2100 equipment operating at 200 kV. The Element mapping of HAADF scanning transmission electron microscopy images were carried out by four symmetrically designed EDS detectors equipped with FEI Talos F200X. PL QY data were measured by an Ocean Optics USB2000 spectrometer equipped with an Ocean Optics ISP-50–8-I integrating sphere. XRD patterns were carried out using Bruker D8-ADVANCE. Transient absorption (TA) spectroscopy measurements were carried out using a Time-Tech Spectra (TA100) transient absorption spectrometer equipped with a 1030 nm femtosecond pulse (10 kHz, 190 fs) and an optical parametric amplifier (ORPHEUS-HE, Light Conversion). The thermal images of devices were measured by a Fluke Ti401 PRO camera. Ultraviolet photoelectron spectroscopy was acquired using a Thermo Scientific ESCALAB 250 XI equipment with a He I discharge lamp (hʋ = 21.22 eV).

The photo-assisted Kelvin probe technique provides a non-contact method for collecting work function differences by measuring the contact potential difference (CPD) between the probe tip and QD. The change of CPD (ΔCPD) with the excitation light, called surface photovoltage, indicates the change in surface band bending. Surface photovoltage spectra were collected by A KP-6500 Digital Kelvin probe system (McAllisterTechnical Services) integrated with a light source (450 W xenon lamp) and a monochromator (IHR 320).

**Device Fabrication**

For the fabrication of QLEDs, the patterned Indium tin oxide (ITO) glass substrates were cleaned by deionized water, acetone, and isopropanol sequentially, then were dried and treated by the UV-ozone process for 15 min. The PEDOT:PSS film was deposited on ITO glass by spin-coated (5000 rpm, 30 s) and annealed at 150 °C for 15 min. Then, these ITO substrates were transferred into an N_2_-atmosphere glove-box. For the TFB layer, the TFB solution (8 mg mL^-1^ in chlorobenzene) was spin-coated onto the substrates at 3000 rpm for 30 s and annealed at 80 °C for 30 min. Subsequently, QD solutions and ZnMgO nanoparticles were spin-coated at 2500 rpm for 30 s and annealed at 60 °C for 30 min. Finally, Al electrodes (100 nm) were deposited by thermal evaporation (∼5 × 10^-6^ mbar). The emitting area of the device was 4 mm^2^ through a shadow mask.

**Device Characterization**

*J-V* curves were performed on an Agilent 4155 C semiconductor parameter analyzer with a calibrated Newport silicon diode under ambient conditions. The electroluminance was measured by a Photo Research spectroradiometer (PR735). An Ocean Optics spectrometer (USB2000) was carried out to obtain the electroluminescence and time resolution electroluminescence spectra and a source meter was used for the calculations of the EQE.

The PCE was calculated according to the following formula:

PCE = *hc*/*q*Vλ*EQE

Where λ is the central wavelength for electroluminescence, *h* is Plank’s constant, *c* is the speed of light in vacuum, *q* is electronic charge, and V is voltage.


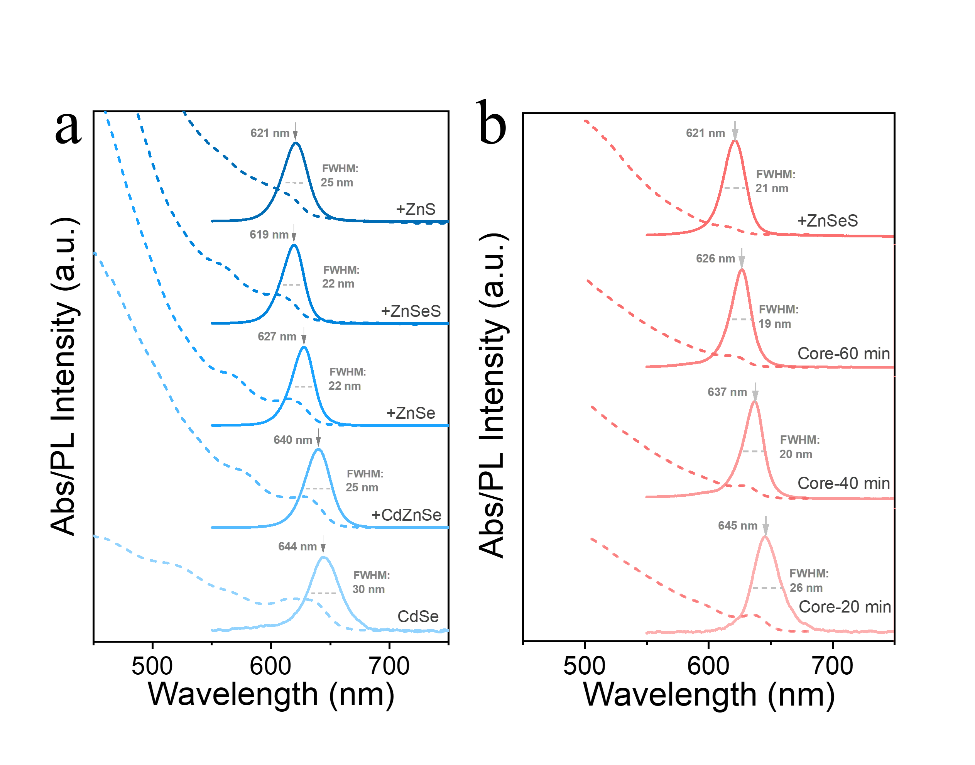


**Fig. S1** (a) The evolution of absorption and PL spectra of CdSe and CdZnSe based QDs.

**Fig. S2** TEM images of (a) CdSe-based QDs and (b) CdZnSe-based QDs.

**Fig. S3** Element mapping of (a-d) CdSe-based QDs and (e-h) CdZnSe-based QDs from HAADF scanning transmission electron microscopy images.

**Fig. S4** Band-edge exciton fine structure of (a) CdSe-based QDs and (b) CdZnSe-based QDs based on absorption spectra.


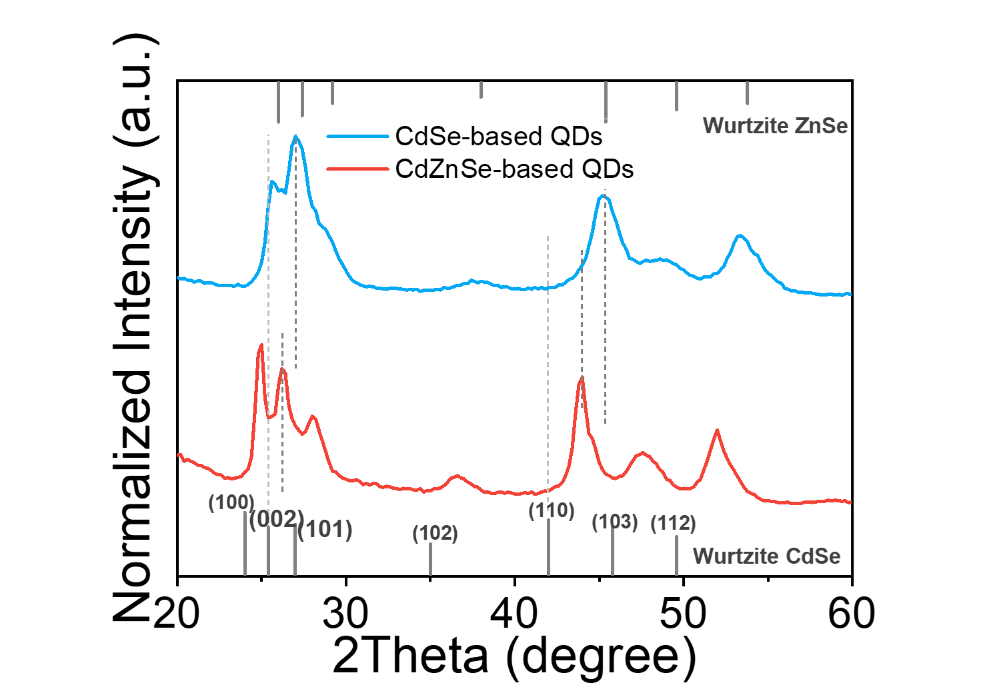


**Fig. S5** XRD patterns for CdSe-based and CdZnSe-based QDs.


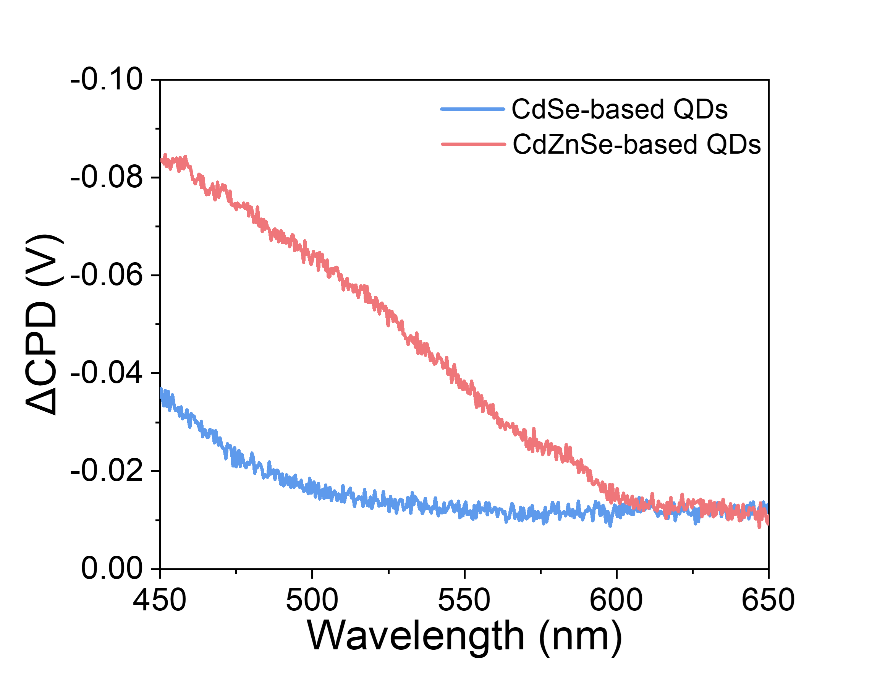


**Fig. S6** Surface photovoltage spectra of CdSe-based QDs and CdZnSe-based QDs.


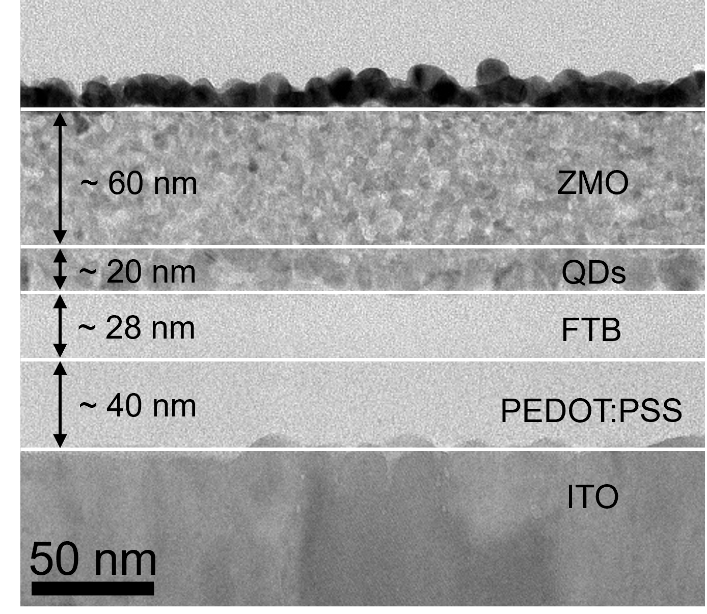


**Fig. S7** Cross-sectional transmission electron microscopy (TEM) image for QLEDs.


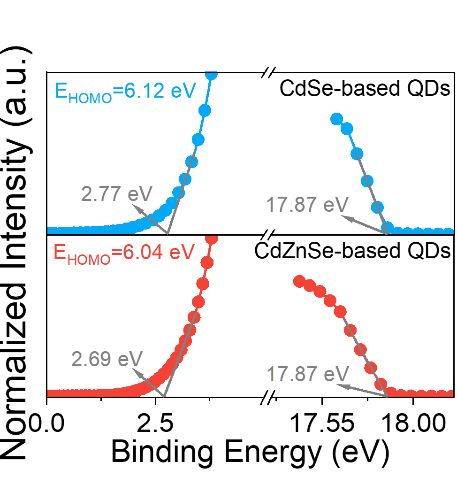


**Fig. S8** UPS spectra for CdSe-based QDs and CdZnSe-based QDs.


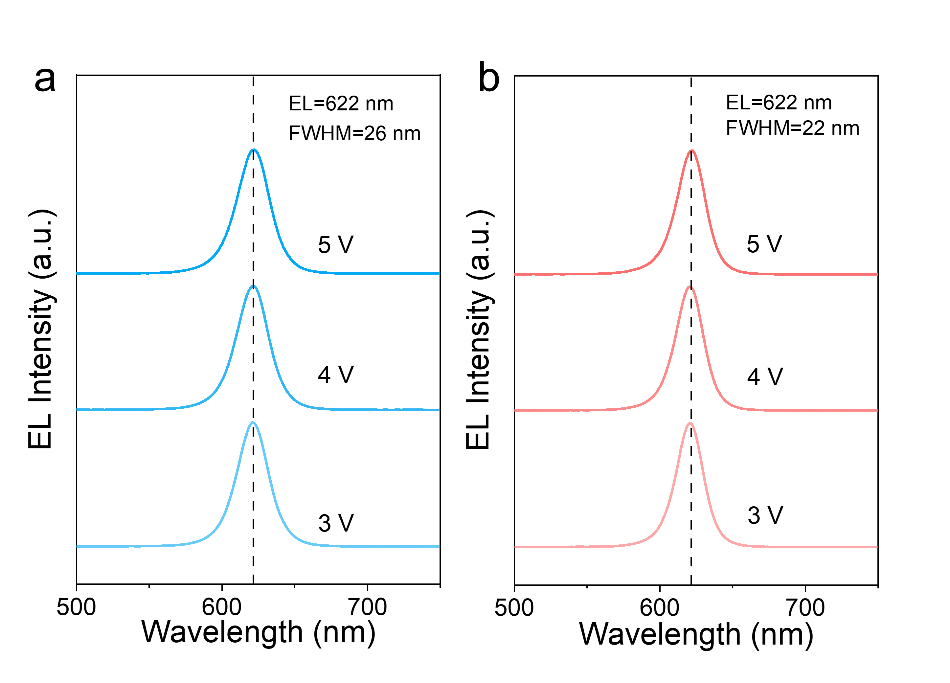


**Fig. S9** EL spectra for QLEDs with CdSe-based QDs and ZnCdSe-based QDs under different bias voltage.

**Table S1** Summary of device parameters for QLED with CdSe-based QDs and CdZnSe-based QDs.

| Devices | *V*_on_ (V) | Maximum EQE (%) | *L* (cd m^-2^) | | Peak PCE (%) | T_95_ (h) @1000 cd m^-2^ |
| --- | --- | --- | --- | --- | --- | --- |
|  |  |  | @bandgap | @120% bandgap |  |  |
| CdSe-based QDs. | 1.65 | 25.8 | ~510 | ~3000 | 20.1 | 20,055 |
| CdZnSe-based QDs. | 1.65 | 28.1 | ~1400 | ~8600 | 27.3 | 72,968 |


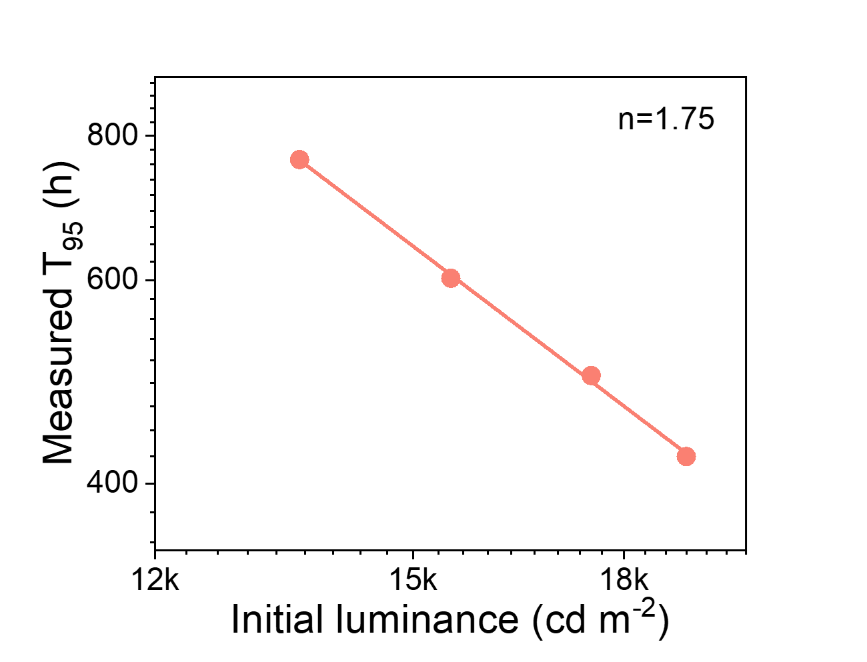


**Fig. S10** T_95_ lifetime measurements at different initial luminance for CdZnSe-based devices. The devices were tested at ambient conditions (temperature, 20-23 °C; relative humidity, 30-40%).


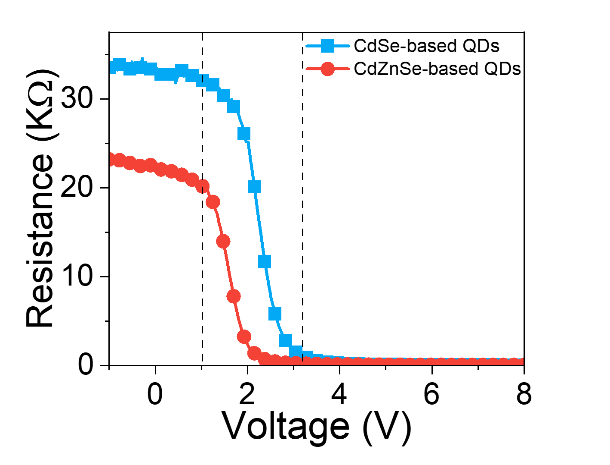


**Fig. S11** Resistance-Voltage curves for QLED with CdSe-based and CdZnSe-based QDs.


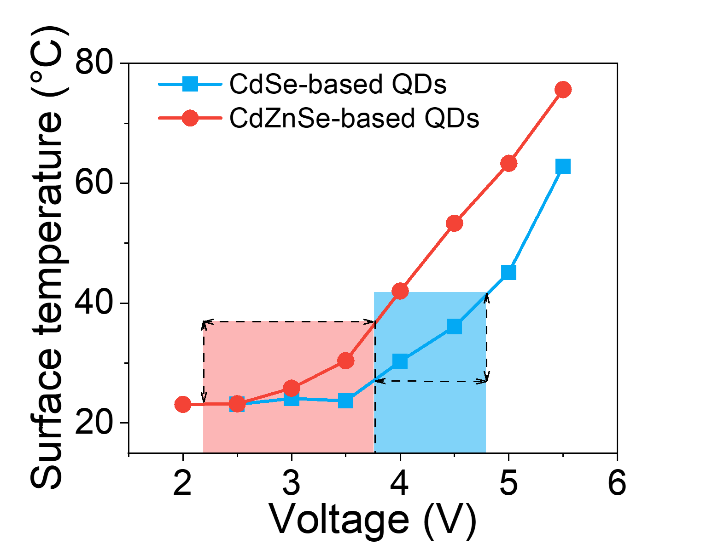


**Fig. S12** Device surface temperature under different voltages at 10 min for CdSe-based and CdZnSe-based QDs.


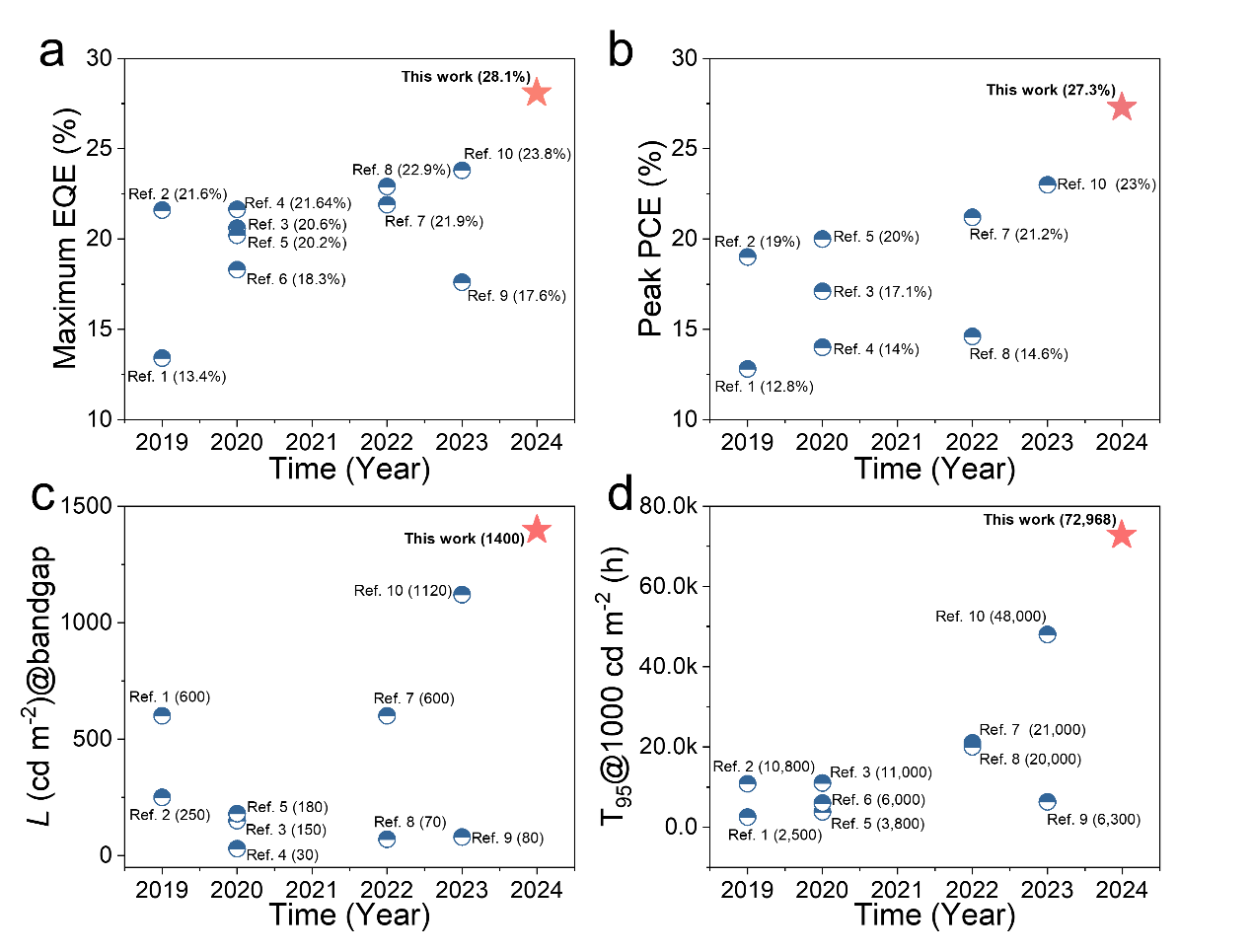


**Fig. S13** Comparison of our devices with other high-performance red QLEDs.

**Table S2** Comparison of our devices with other high-performance red QLEDs.

|  | Year | Maximum EQE (%) | Peak PCE (%) | T_95_@ 1000 cd m^-2^ (h) | *L* (cd m^-2^) @bandgap | Reference |
| --- | --- | --- | --- | --- | --- | --- |
| 1 | 2019 | 13.4 | 12.8 | 2,500 | ~600 | ^1^Adv. Funct. Mater. 2020, 30, 1907265 |
| 2 | 2019 | 21.6 | 19 | 10,800 | ~250 | ^2^Nat. Photonics 2019, 13, 192–197 |
| 3 | 2020 | 20.6 | 17.1 | ~11,000 | ~150 | ^3^J. Phys. Chem. Lett. 2020, 11, 3111−3115 |
| 4 | 2020 | 21.64 | 14.0 | - | ~30 | ^4^Adv. Optical Mater. 2020, 8, 1902092 |
| 5 | 2020 | 20.2 | 20 | 3,800 | ~180 | ^5^Nat. Commun. 2020, 11, 937 |
| 6 | 2020 | 18.3 | - | ~6,000 | - | ^6^ACS Nano 2020, 14, 17496-17504 |
| 7 | 2022 | 21.9 | ~21.2 | 21,000 | ~600 | ^7^Adv. Funct. Mater. 2022, 2207974 |
| 8 | 2022 | 22.9 | 14.6 | 20,000 | 70 | ^8^Nano Lett. 2023, 23, 6689-6697 |
| 9 | 2023 | 17.6 | - | ~6300 | ~80 | ^9^Nano Lett. 2023, 23, 1061-1067 |
| 10 | 2023 | 23.8 | 23.0 | 48,000 | 1,120 | ^10^Nat. Nanotechnol. 2023, 18, 1168-1174 |
| 11 | **2024** | **28.1** | **27.3** | **72,968** | **1,400** | **This work** |

Reference

1. Lin, J.; Dai, X.; Liang, X.; Chen, D.; Zheng, X.; Li, Y.; Deng, Y.; Du, H.; Ye, Y.; Chen, D.; Lin, C.; Ma, L.; Bao, Q.; Zhang, H.; Wang, L.; Peng, X.; Jin, Y., High-performance quantum-dot light-emitting diodes using NiO hole-injection layers with a high and stable work function. *Adv. Funct. Mater.* **2020,** *30* (5), 1907265.

2. Shen, H.; Gao, Q.; Zhang, Y.; Lin, Y.; Lin, Q.; Li, Z.; Chen, L.; Zeng, Z.; Li, X.; Jia, Y.; Wang, S.; Du, Z.; Li, L. S.; Zhang, Z., Visible quantum dot light-emitting diodes with simultaneous high brightness and efficiency. *Nat. Photonics* **2019,** *13* (3), 192-197.

3. Liu, D.; Cao, S.; Wang, S.; Wang, H.; Dai, W.; Zou, B.; Zhao, J.; Wang, Y., Highly stable red quantum dot light-emitting diodes with long T_95_ operation lifetimes. *J. Phys. Chem. Lett.* **2020,** *11* (8), 3111-3115.

4. Zhang, H.; Su, Q.; Chen, S., Suppressing Förster resonance energy transfer in close-packed quantum-dot thin film: Toward efficient quantum-dot light-emitting diodes with external quantum efficiency over 21.6%. *Adv. Opt. Mater.* **2020,** *8* (10), 1902092.

5. Pu, C.; Dai, X.; Shu, Y.; Zhu, M.; Deng, Y.; Jin, Y.; Peng, X., Electrochemically-stable ligands bridge the photoluminescence-electroluminescence gap of quantum dots. *Nat. Commun.* **2020,** *11* (1), 937.

6. Rhee, S.; Chang, J. H.; Hahm, D.; Jeong, B. G.; Kim, J.; Lee, H.; Lim, J.; Hwang, E.; Kwak, J.; Bae, W. K., Tailoring the electronic landscape of quantum dot light-emitting diodes for high brightness and stable operation. *ACS Nano* **2020,** *14* (12), 17496-17504.

7. Cheng, Y.; Gui, Z.; Qiao, R.; Fang, S.; Ba, G.; Liang, T.; Wan, H.; Zhang, Z.; Liu, C.; Ma, C.; Hong, H.; Fan, F.; Liu, K.; Shen, H., Electronic structural insight into high-performance quantum dot light-emitting diodes. *Adv. Funct. Mater.* **2022,** *32* (48), 2207974.

8. Liu, X.; Wang, L.; Gao, Y.; Zeng, Y.; Liu, F.; Shen, H.; Manna, L.; Li, H., Ultrastable and high-efficiency deep red QLEDs through giant continuously graded colloidal quantum dots with shell engineering. *Nano Lett.* **2023,** *23* (14), 6689-6697.

9. Chen, D.; Ma, L.; Chen, Y.; Zhou, X.; Xing, S.; Deng, Y.; Hao, Y.; Pu, C.; Kong, X.; Jin, Y., Electrochemically stable ligands of ZnO electron-transporting layers for quantum-dot light-emitting diodes. *Nano Lett.* **2023,** *23* (3), 1061-1067.

10. Gao, Y.; Li, B.; Liu, X.; Shen, H.; Song, Y.; Song, J.; Yan, Z.; Yan, X.; Chong, Y.; Yao, R.; Wang, S.; Li, L. S.; Fan, F.; Du, Z., Minimizing heat generation in quantum dot light-emitting diodes by increasing quasi-Fermi-level splitting. *Nat. Nanotechnol.* **2023,** *18* (10), 1168-1174.
